# The Chain Mediating Effect of Instrumental Activities of Daily Living and Regular Exercise in the Relationship Between Physical Performance and Cognitive Function Among Older Adults Comorbid With Diabetes Mellitus and Hypertension

**DOI:** 10.1002/brb3.71305

**Published:** 2026-03-23

**Authors:** Chao Sun, Huixiu Hu, Xiaoyan Zhang, Yajie Zhao, Wei Gu

**Affiliations:** ^1^ Department of Nursing, Beijing Hospital, National Center of Gerontology, Institute of Geriatric Medicine Chinese Academy of Medical Sciences Beijing China; ^2^ School of Economics and Management University of Science and Technology Beijing Beijing P.R. China; ^3^ Department of Vascular Surgery, Beijing Hospital, National Center of Gerontology, Institute of Geriatric Medicine Chinese Academy of Medical Sciences Beijing P.R. China; ^4^ Department of Cardiology, Beijing Hospital, National Center of Gerontology, Institute of Geriatric Medicine Chinese Academy of Medical Sciences Beijing P.R. China

**Keywords:** cognitive function, diabetes, hypertension, instrumental activities of daily living, older adult, physical performance, regular exercise

## Abstract

**Purpose:**

To develop a chain mediation model to elucidate the relationship among physical performance, instrumental activities of daily living (IADL), regular exercise, and cognitive function among older adults who are comorbid with diabetes mellitus and hypertension (OA‐DM&HTN).

**Methods:**

A total of 656 participants were investigated with the Mini‐Mental State Examination, the Short Physical Performance Battery, the Instrumental Activities of Daily Living, and a questionnaire on regular exercise frequency between January and September 2022. Sequential multiple mediation models were conducted to analyze the data.

**Results:**

The average age of the participants was 73.47 ± 7.40 years, and 49.24% (*n* = 323) of participants were female. The average cognitive function score was 22.36 ± 6.14, and 32.62% (*n* = 214) of participants exhibited cognitive impairment. Cognitive performance exhibited significant associations with demographic factors, including gender, age, marriage status, educational background, and income level (*p* < 0.05). Chain mediation analysis indicated that physical performance directly predicted cognitive function (*β* = 0.525, *95% CI*: 0.000–1.050); physical performance had indirect effects mediated by IADL (*β* = 0.917, 95% CI: 0.635–1.230) and regular exercise (*β* = 0.076, 95% CI: 0.003–0.180). A significant chain‐mediating effect involving both IADL and regular exercise was also observed on the relationship between physical performance and cognitive function (*β* = 0.034, 95% CI: 0.002–0.071).

**Conclusion:**

Physical performance is a significant predictor of cognitive function, and it can also affect cognitive function through the independent or chain‐mediating effects of IADL and regular exercise among OA‐DM&HTN. Therefore, to delay cognitive decline among OA‐DM&HTN, it is essential to provide tailored functional training, encourage improvement in IADL, and promote regular exercise among OA‐DM&HTN.

## Graphical abstract

This study established a chain mediation model among older adults comorbid with diabetes mellitus and hypertension (n = 656). Physical performance was directly associated with cognitive function and was also indirectly linked with it through instrumental activities of daily living (IADL) and regular exercise as independent mediators, as well as through their sequential pathway. These findings highlight multifaceted intervention targets—functional training, IADL improvement, and exercise promotion—to delay cognitive decline in this vulnerable population.

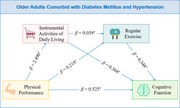

## 1 Introduction

With the global population aging, the coexistence of diabetes and hypertension has become increasingly prevalent among the elderly. Based on the data provided by the World Health Organization (WHO), around 40% of older adults worldwide have hypertension, while the incidence of diabetes is particularly notable in individuals aged 65 years and above (World Health Organization, Chang and Mingsheng 2024). A population‐oriented longitudinal investigation (Chen et al. 2023) revealed that individuals with poor glycemic regulation demonstrated a 54% increased risk of developing hypertension relative to those maintaining optimal glucose levels. Furthermore, when juxtaposed with non‐hypertensive individuals who had a low propensity score, hypertensive patients possessing a high propensity score exhibited a 2.646‐fold elevation in the risk of newly‐onset diabetes. This was evidenced by a hazard ratio of 3.646, with a *p*‐value of less than 0.0001 (Wu et al. 2021). These findings collectively demonstrate the bidirectional relationship and synergistic effects between diabetes and hypertension in older populations.

The coexistence of diabetes and hypertension significantly impairs cognitive function in older adults (Christian et al. 2024; Wang et al. 2023). Hyperglycemia and insulin resistance in diabetes contribute to neuronal injury and cerebrovascular impairment through sustained metabolic dysregulation, exacerbated by oxidative stress and chronic inflammatory processes (Garcia‐Serrano and Duarte 2020; Teodoro et al. 2018; Zhang et al. 2023). Ashebir et al. reported that a majority of diabetic individuals exhibit cognitive dysfunction, with a prevalence rate of 56.3% (Ashebir et al. 2024). Meanwhile, hypertension promotes chronic cerebral hypoperfusion and microvascular damage, disrupting cerebral hemodynamics and impairing neurovascular coupling—key mechanisms underpinning cognitive deterioration (Katsi et al. 2024). Chronic inflammation represents a common pathological pathway linking both conditions; pro‐inflammatory cytokines such as interleukin‐6 exacerbate endothelial dysfunction and impair neuroplasticity, thereby compounding cognitive decline (Marioni et al. 2010). Older adults comorbid with diabetes mellitus and hypertension (OA‐DM&HTN) experience more pronounced cognitive deterioration than those with single conditions (Marioni et al. 2010). Therefore, early identification of factors affecting cognitive function and development of targeted interventions are essential for mitigating cognitive decline in this population.

Chronic hyperglycemia causes complications that compromise physical function through peripheral neuropathy, myopathy, and advanced glycation end‐products (Lopatin et al. 2025; Minata et al. 2024). Hypertension further deteriorates performance via cardiovascular deconditioning and cerebrovascular changes (Tunc Suygun et al. 2025). When coexisting, these conditions synergistically accelerate physical decline through vascular dysfunction, inflammation, and sarcopenia. Recent studies investigating physical performance—encompassing balance, gait velocity, and muscular strength—demonstrate notable linkages with cognitive functioning among OA‐DM&HTN (Wu et al. 2021). Cross‐sectional research showed that individuals with higher dynamic balance capabilities exhibited enhanced cognitive function, with cognitively healthy participants scoring 2.526 points higher on physical performance assessments (Komalasari et al. 2024). Another study of 13,716 participants revealed significant correlations between cognitive function scores and grip strength as well as chair‐standing time (Ma et al. 2024). Reduced gait velocity and elevated blood glucose levels correlate with cognitive impairments, particularly in executive functioning (Umegaki et al. 2017). Physical capability has been acknowledged as an independent prognostic factor for cognitive deterioration (Umegaki et al. 2017; David et al. 2015). Consequently, drawing upon the existing body of evidence, the following hypothesis is put forward: physical performance acts as a crucial prognostic indicator for cognitive function among OA‐DM&HTN.

Research has shown that older adults suffering from chronic comorbidities are more likely to experience impairments in instrumental activities of daily living (IADL) (Geng et al. 2022; Ribeiro‐Lucas et al. 2025). IADL impairments significantly reduce social participation, disrupting socio‐cognitive integration and triggering adverse health outcomes, including accelerated cognitive decline (Bae 2020). Studies demonstrate that IADL limitations inversely correlate with cognitive performance metrics, while reduced social engagement exacerbates cognitive frailty (Bae 2020). In patients with chronic comorbidities, IADL impairments often accompany disuse‐related physical function decline, exacerbating health challenges and increasing cognitive impairment risk (Zeng et al. 2017; Feng et al. 2024). Declining physical performance directly impacts IADL capacity—reduced balance may impair shopping or household activities, limiting self‐management and independent living. The synergistic effects of IADL impairments and declining physical performance may accelerate cognitive decline through reduced social engagement and cognitive stimulation opportunities. Based on this evidence, we propose Hypothesis 2: IADL acts as an intermediary mechanism linking physical performance and cognitive function among OA‐DM&HTN.

Moreover, better physical performance reflects higher cardiovascular adaptability and muscle strength, supporting higher‐intensity aerobic exercise—a critical factor influencing cognitive function. Cross‐sectional analysis revealed that time spent on physical activities positively associates with cognitive performance in older adults (Wu et al. 2024). Regular exercise elicits neurovascular adaptations that enhance cerebral blood circulation, especially in brain regions vital for cognitive processing. Sustained aerobic activity improves neurovascular coupling within the hippocampus and prefrontal cortex, facilitating synaptic plasticity, neurogenesis, and metabolic efficiency that enhance episodic memory, working memory, and decision‐making (Jeyarajan et al. 2024; So et al. 2024). This process involves improved angiogenesis and cerebral blood flow regulation, which is particularly beneficial for addressing cerebral hypoperfusion caused by metabolic disorders and vascular stiffness in OA‐DM&HTN. Based on these findings, we posit Hypothesis 3: regular exercise serves as a mechanistic mediator linking physical performance and cognitive function among OA‐DM&HTN.

**FIGURE 1:**
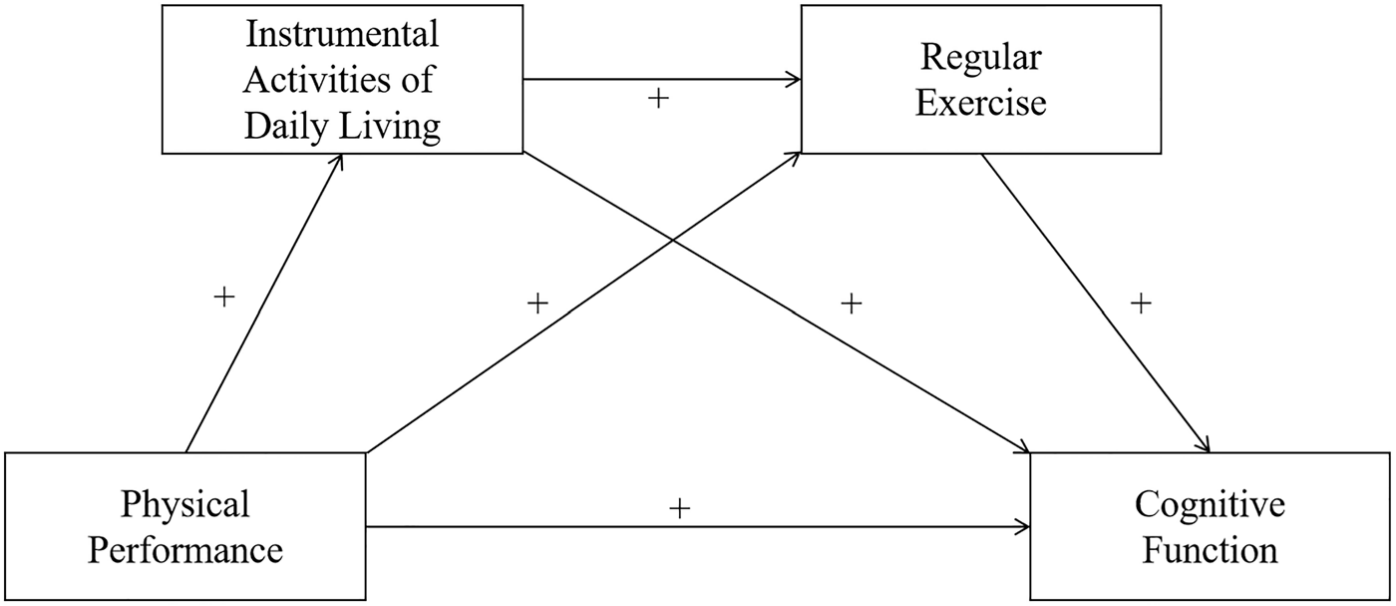
Hypothetical graph.

Additionally, IADL and regular exercise are closely related. IADL capacity directly influences the motivation and capability of OA‐DM&HTN to maintain regular exercise habits. OA‐DM&HTN individuals with higher IADL performance show increased participation in structured exercise and exhibit stronger initiative in self‐care and health enhancement. Building upon the observed associations, we advance Hypothesis 4: IADL and regular exercise synergistically form a sequential mediation pathway that translates physical performance into cognitive function among OA‐DM&HTN (Figure 1). This study will test the proposed hypotheses.

## 2 Materials and Methods

### 2.1 Study Design

A cross‐sectional analysis was conducted to explore the potential relationships and sequential mediating effects of IADL and regular exercise on the association between physical performance and cognitive function among OA‐DM&HTN. This study followed the Strengthening the Reporting of Observational Studies in Epidemiology (STROBE) reporting guideline (Von Elm et al. 2008).

### 2.2 Data Collection

#### 2.2.1 Participants

A multi‐stage convenience sampling approach was employed to select research sites, encompassing multiple community health centers and hospitals across Fujian and Jiangsu provinces in China. This sampling strategy was designed to capture participants with varying disease severity and socioeconomic backgrounds, as patients typically seek care at different healthcare levels based on their condition complexity and accessibility. The survey was conducted between January and September 2022. Sample size determination was conducted using the formula for estimating sample size in cross‐sectional surveys: $n={\left(\frac{Z_{\alpha /2}}{\delta }\right)}^{2}\;P\;\left(1-P\right)$where *Z_α/2_* = 1.96 (95% CI), the allowable margin of error was set at 5%, and *p* = 30% based on previous studies (Shi et al. 2021). The estimated necessary sample size was determined to be 323 participants. A 20% buffer was added to the initial sample size to account for unusable data, resulting in a minimum final sample of 388. Inclusion criteria: diagnosed with diabetes mellitus combined with hypertension by a hospital; aged 60 years and over; good communication skills. Exclusion criteria: diagnosed with psychiatric disorders (e.g., major depressive disorder); inability to cooperate with physical function assessments (e.g., due to complete physical disability); currently experiencing acute illness exacerbation (e.g., post‐surgical recovery, acute infection, or critical illness requiring intensive medical intervention). The study protocol received approval from the Ethics Committee of Beijing Hospital (Approval No. 2021BJYYEC‐325‐01).

### 2.3 Measures

#### 2.3.1 Cognitive Function Assessment

The cognitive function of OA‐DM&HTN was assessed using the Mini‐Mental State Examination (MMSE), developed by Folstein and colleagues (Folstein et al. 1975). The MMSE serves as a tool for assessing multiple cognitive dimensions. These encompass orientation regarding time and location, immediate and long‐term memory retrieval, attentiveness and computational skills, linguistic proficiency, and visuospatial perception. The overall score on the MMSE spans from 0 to 30, where a higher score is indicative of higher cognitive function. The criteria for classifying normal cognitive function are defined as follows: for individuals with a middle‐school education or above, a score greater than 24; for those with a primary‐school education, a score exceeding 20; and for illiterate individuals, a score above 17. The presence of cognitive impairment is suggested by scores falling below these respective benchmarks. The Chinese‐adapted version of the MMSE demonstrated a test‐retest reliability of 0.91 (Wang and Zhang 1989).

#### 2.3.2 Physical Performance Assessment

The physical performance of OA‐DM&HTN was assessed using the Short Physical Performance Battery (SPPB), a well‐established instrument created by the US National Institute on Aging. It consists of three components that measure balance, gait speed, and lower extremity muscle strength. Balance Test: It involves three standing postures: feet‐together, semi‐tandem, and tandem. Scoring criteria are as follows: standing for > 10 s in feet‐together and semi‐tandem positions earns 1 point; standing for 3–<10 s in the tandem position earns 1 point, while standing for 10 s earns 2 points. Gait Speed Test: This involves a 2.44‐meter walking speed test. Scores are assigned based on walking speed: 1 point is assigned for speeds < 0.43 m/s, 2 points for speeds between 0.44 and 0.60 m/s, 3 points for speeds between 0.61 and 0.77 m/s, and 4 points for speeds ≥ 0.78 m/s. Muscle Strength Test: This is assessed using a 5‐repetition chair stand test. Scoring is as follows: 16.7–60 s (1 point), 13.70–16.69 (2 points), 11.20–13.69 (3 points), and ≤ 11.19 s (4 points). Each component of the assessment is scored from 0 to 4, with higher scores indicating superior physical performance. A total score < 3 indicates functional impairment (Podsiadlo and Richardson 1991).

#### 2.3.3 Instrumental Activities of Daily Living Assessment

The IADL of OA‐DM&HTN was evaluated using the Instrumental Activities of Daily Living scale, formulated by Lawton and colleagues (Lawton and Brody 1969). We specifically selected IADL rather than basic ADL because IADL is a more sensitive indicator of early functional decline and requires advanced cognitive abilities, including executive function, planning, and problem‐solving. The scale measures IADL competence across eight domains, producing a total score ranging from 0 to 24. Higher scores reflect better IADL performance, with a maximum possible score of 24. The IADL scale has gained widespread international adoption and exhibits satisfactory reliability and validity.

#### 2.3.4 Regular Exercise Assessment

In accordance with the WHO guidelines, individuals should accumulate a minimum of 150 min per week of moderate‐to‐vigorous intensity physical activity (MVPA), preferably spread throughout the week to support sustained engagement in regular exercise and optimize health outcomes (Bull et al. 2020). As defined by the WHO (Bull et al. 2020), moderate‐intensity physical activity (MPA) refers to activities performed at an intensity ranging from 3.0 to 5.9 metabolic equivalents (METs), which generally corresponds to a perceived exertion level rated as 5 or 6 on a scale of 0 to 10, accounting for individual fitness levels. Vigorous‐intensity physical activity (VPA) was defined as activities performed at ≥ 6.0 METs, usually corresponding to a rating of 7 or 8 on the same perceived exertion scale relative to individual capacity. The frequency of regular exercise for OA‐DM&HTN can be categorized into four levels: no exercise (assigned a score of 1), 1–3 days/week (score = 2), 4–6 days/week (score = 3), and daily exercise (score = 4). For each category, exercise sessions were required to comprise a minimum of 20 min of moderate‐intensity exercise or an equivalent duration of high‐intensity exercise per day to constitute a clinically relevant dose. Activities lasting less than 10 min are not considered sufficient to count towards the total.

#### 2.3.5 Covariates

To control for potential confounding factors, sociodemographic variables, health‐related behaviors, and clinical characteristics were considered for inclusion as covariates in the analysis. Sociodemographic factors encompassed age, gender, body mass index (BMI), marital status, educational background, and monthly per capita household income. Health behaviors encompassed smoking status and alcohol use. Clinical covariates involved documented histories of coronary heart disease, cerebrovascular disease, as well as polypharmacy, which was defined as the regular use of five or more medications (Wu et al. 2025).

### 2.4 Data Collection Process

In this study, evaluators were designated at each study site to supervise the data‐gathering process. The data collection was carried out via the “Jingyi Elderly Function Assessment Platform” WeChat mini‐program, which was developed by the research consortium. To guarantee the standardized utilization of the platform, the research team offered a unified training program to all evaluators from the participating sites through virtual conferences. Before data collection, the evaluators utilized standardized protocols to clarify the study objectives and methodologies to participants, thereby ensuring that informed consent was obtained before commencing the data‐gathering process. To maintain data quality, the researchers conducted a weekly review of the questionnaire completion durations through the platform's backend. They specifically scrutinized questionnaires completed between 10:00 PM and 8:00 AM or within a time frame of less than 15 min to verify the authenticity of the data. During the data‐cleansing phase, questionnaires were excluded if they contained missing data from the scales, more than 15% missing demographic details, uniform or wave‐shaped response patterns, or logical inconsistencies.

### 2.5 Statistical Analysis

All statistical analyses were performed using IBM SPSS Statistics version 25.0 (SPSS Inc., Chicago, IL, USA). The significance level was set as 0.05. Differences in cognitive function scores across groups defined by sociodemographic, health behavior, and clinical variables were evaluated using the independent samples *t*‐test and analysis of variance. Continuous variables are summarized as mean ± standard deviation, and categorical variables are reported as frequencies and percentages. The strength and direction of associations between variables were quantified using Pearson's correlation coefficient or Spearman's rank correlation coefficient, depending on data distribution. Multicollinearity was assessed by calculating the variance inflation factor (VIF) (Kim 2019). The VIF for all covariates was well below the common threshold of 5 (with a maximum VIF of 1.300), indicating that multicollinearity was not an issue in the model. The PROCESS macro, developed by Hayes (2018), was used to assess the statistical significance of mediation effects. This approach integrates ordinary least squares (OLS) regression and the bootstrap method for robust estimation. Following Hayes' instructions, bootstrap analyses were carried out using the IBM SPSS PROCESS macro, which was created by Andrew F. Hayes and ran Serial Multiple Mediation Model 6 (Hayes 2013). Over 5000 bootstrap samples, the mediating variable's statistical significance was investigated. This approach provided an estimate of the indirect effect along with its 95% CI. The indirect effect was statistically significant (*p* < 0.05), as indicated by a 95% CI that did not include zero.

To examine the hypothesized mediating pathways, a sequential multiple mediation model was established using the PROCESS macro v3.4. In this model, physical performance served as the independent variable, cognitive function as the dependent variable, and both IADL and regular exercise were included as serial mediators. All analyses were adjusted for baseline variables that demonstrated significant differences between groups to control for potential confounding factors.

## 3 Results

### 3.1 Baseline Characteristics of the Participants

Of the 656 participants included in this study, 49.24% (*n* = 323) were female (Table 1). The mean age was 73.47 ± 7.40 years, with the majority (58.69%, *n* = 385) falling within the 60–74 years age range. Most participants (63.41%, *n* = 416) had a primary school education or below. The mean cognitive function score was 22.36 ± 6.14 (range 0–30). Based on established MMSE cutoffs, 32.62% (*n* = 214) of participants exhibited cognitive impairment. Cognitive function scores varied significantly by gender, age group, marital status, educational background, and income level (all *p* < 0.05). In contrast, no significant differences were observed for BMI, smoking, alcohol consumption, comorbid coronary heart disease, comorbid cerebrovascular disease, and polypharmacy status (all *p* > 0.05). Furthermore, physical performance scores averaged 1.54 ± 0.78 (range 0–12). IADL scores averaged 17.37 ± 6.19 (range 0–24). Regarding regular exercise patterns, the frequency distribution revealed that no exercise was the most prevalent category (*n* = 205, 31.25%), followed by 1–3 days per week (*n* = 184, 28.05%), and daily exercise (*n* = 175, 26.68%), with a median frequency of 2 (interquartile range = 1–4).

**TABLE 1:** Score differences of each variable by demographic characteristics (*n* = 656).

Characteristic	Categories	Number of cases (%)	Cognitive function scores $\bar{x}\pm s$	*F/t*	*P*
Gender	Male	333 (50.76)	23.67 ± 5.96	5.711	<0.001*
	Female	323 (49.24)	21.00 ± 6.02		
Age	60∼74	385 (58.69)	23.58 ± 5.27	17.885	<0.001*
	75∼89	261 (39.79)	20.81 ± 6.65		
	≥ 90	10 (1.52)	15.7 ± 9.82		
BMI (kg/m^2^)	< 18.5	39 (5.95)	21.85 ± 5.38	0.231	0.794
	18.5∼23.9	319 (48.62)	22.49 ± 6.15		
	> 24	298 (45.43)	22.28 ± 6.23		
Education level	Primary school or below	416 (63.41)	20.33 ± 5.99	101.683	<0.001*
	Junior middle school	124 (18.90)	24.72 ± 5.18		
	Senior high school or vocational school	88 (13.41)	26.69 ± 3.85		
	College/associate degree or higher	28 (4.27)	28.32 ± 2.21		
Marital status	Married	555 (84.60)	22.76 ± 5.95	3.995	<0.001*
	Others	101 (15.40)	20.14 ± 6.69		
Income (RMB/Month)	< 5000	534 (81.40)	21.77 ± 6.18	−5.836	<0.001*
	≥ 5000	122 (18.60)	24.94 ± 5.24		
Coronary heart disease	No	523 (79.72)	22.32 ± 6.11	−0.341	0.733
	Yes	133 (20.27)	22.52 ± 6.25		
Cerebrovascular disease	No	608 (92.68)	22.46 ± 6.10	1.470	0.142
	Yes	48 (7.32)	21.10 ± 6.50		
Polypharmacy	No	397 (60.52)	22.23 ± 6.15	−0.659	0.510
	Yes	259 (39.48)	22.55 ± 6.12		
Smoking	No	503 (76.68)	22.38 ± 6.18	0.144	0.886
	Yes	153 (23.32)	22.29 ± 6.00		
Alcohol consumption	No	493 (75.15)	22.38 ± 6.16	0.164	0.870
	Yes	163 (24.85)	22.29 ± 6.07		
Registration location	Community	118 (17.99)	22.07 ± 5.99	−0.565	0.573
	Hospital	538 (2.01)	22.42 ± 6.17		

*Note*: ^*^: *p* < 0.05.

### 3.2 Common Method Bias Test

Common method variance was assessed using Harman's single‐factor test, in which all items from the measurement instruments were subjected to exploratory factor analysis. The analysis identified nine factors with eigenvalues greater than 1. The largest factor accounted for 27.36% of the total variance, which is below the recommended threshold of 40%. These results indicate that common method bias was not a significant concern in this study.

### 3.3 Correlation Analysis

The findings from the correlation analysis demonstrated a significant positive association between physical performance and cognitive function (*r* = 0.341, *p* < 0.001), IADL (*r* = 0.358, *p* < 0.001), and regular exercise (*r* = 0.278, *p* < 0.001). Additionally, IADL was positively correlated with regular exercise (*r* = 0.291, *p* < 0.001) and cognitive function (*r* = 0.476, *p* < 0.001). Regular exercise demonstrated a statistically significant positive relationship with cognitive function *(r* = 0.282, *p* < 0.001). See Table 2 for more details.

**TABLE 2:** Analysis of the correlation between physical performance, instrumental activities of daily living, regular exercise, and cognitive function (*n* = 656).

Variable	PP	IADL	RE	CF
**PP**	—	—	—	—
**IADL**	0.358**^a^	—	—	—
**RE**	0.278**^b^	0.291**^b^	—	—
**CF**	0.341**^a^	0.476**^a^	0.282**^b^	—

*Note*: **p < 0.001; a: Pearson correlation coefficients; b: Spearman correlation coefficients; Physical performance: PP; Instrumental activities of daily living: IADL; Regular exercise: RE; Cognitive function: CF.

### 3.4 Chain Mediation Model Regression Analysis Results

**FIGURE 2:**
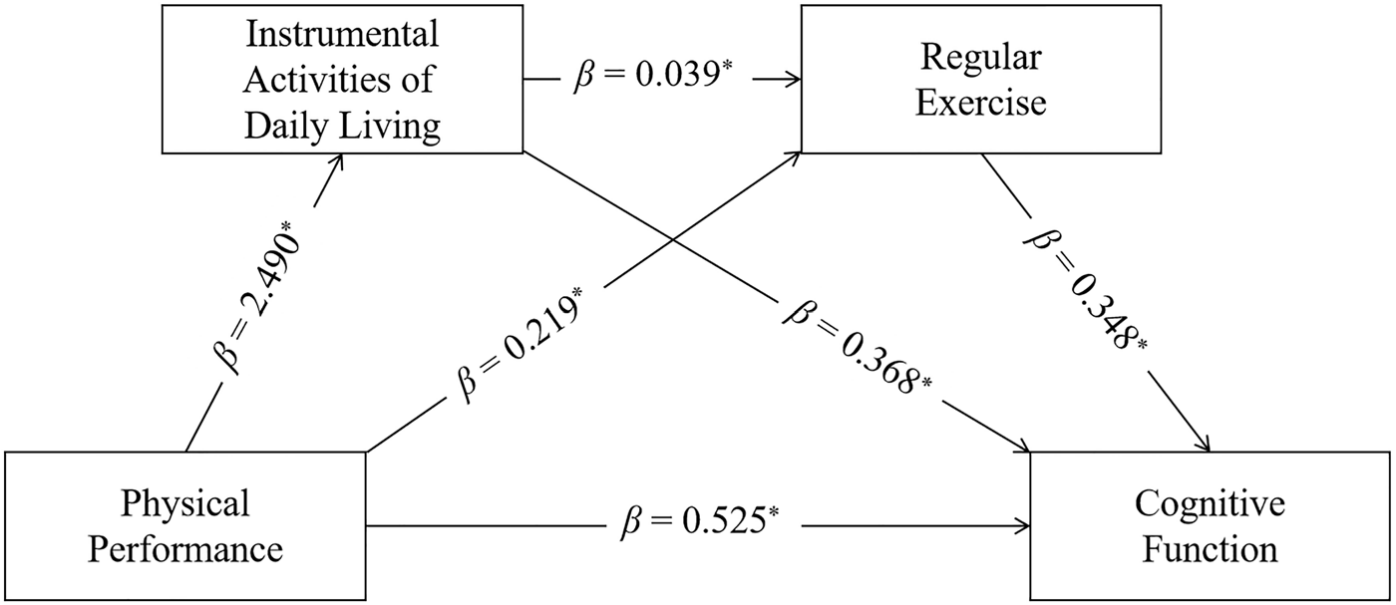
Diagram of the chain mediation model.

Based on descriptive statistics and correlation outcomes, this research established a chain mediation model examining the relationships between physical performance (independent variable), cognitive function (dependent variable), and the sequential mediators of IADL and regular exercise. The analysis incorporated adjustments for age, gender, educational background, marital status, and mean monthly household income. The results indicated that physical performance positively predicted IADL (*β* = 2.490, *p* < 0.05), regular exercise (*β* = 0.219, *p* < 0.05), and cognitive function (*β* = 0.525, *p* < 0.05). Similarly, IADL showed a positive association with regular exercise (*β* = 0.039, *p* < 0.05) and cognitive function (*β* = 0.368, *p* < 0.05). Additionally, regular exercise significantly and positively influenced cognitive function (*β* = 0.348, *p* < 0.05). More details can be seen in Table 3 and Figure 2.

**TABLE 3:** Chain mediation model regression analysis results (*n* = 656).

Variable	Model 1: IADL			Model 2: RE			Model 3: CF		
	*coeff*	*Se*	*t*	*coeff*	*Se*	*t*	*coeff*	*Se*	*t*
**Constant**	15.952	1.382	11.547*	1.475	0.299	4.935*	13.394	1.300	10.301*
**PP**	2.490	0.299	8.329*	0.219	0.062	3.537*	0.525	0.267	1.965*
**IADL**	—	—	—	0.039	0.008	5.017*	0.368	0.033	10.931*
**RE**	—	—	—	—	—	—	0.348	0.168	2.072*
**Gender**	1.125	0.464	2.424*	−0.133	0.092	−0.145	−1.266	0.393	−3.22*
**Age**	−2.961	0.434	−6.818*	−0.198	0.087	−2.235*	−9.953	0.380	−2.511*
**Education level**	0.300	0.272	1.101	0.100	0.054	1.865	2.304	0.230	10.019*
**Marital Status**	−0.092	0.313	−0.295	−0.045	0.062	−0.727	−0.406	0.264	−1.541
**Income**	−0.174	0.591	−0.294	0.216	0.117	1.856	0.905	0.499	1.812
**Source**									
**Hyperlipidemia**									
***R* ^2^**	0.2007			0.148			0.427		
***F***	27.157*			16.080*			60.139*		

*Note*: *p < 0.05; Physical performance: PP; Instrumental activities of daily living: IADL; Regular exercise: RE; Cognitive function: CF.

### 3.5 Chain Mediation Path Analysis

Physical performance showed a statistically significant direct influence on cognitive function in OA‐DM&HTN (*β* = 0.525, 95% CI: 0.000–1.050). The independent mediating effect of IADL was also significant (*β* = 0.917, 95% CI: 0.635–1.230), as was the independent mediating effect of regular exercise (*β* = 0.076, 95% CI: 0.003–0.180). Moreover, the chain mediation pathway involving both IADL and regular exercise was statistically significant in its influence on cognitive function (*β* = 0.034, 95% CI: 0.002–0.071). These results indicate that physical performance affects cognitive function via a sequential mechanism that includes both IADL and regular exercise. Additional details are provided in Table 4.

**TABLE 4:** Chain mediation path analysis (*n* = 656).

Path	Effect	Boot SE	Bootstrap95%CI		Proportion of effect(%)
			BootLLCI	BootULCI	
**Total effect**	1.552	0.277	1.008	2.095	100.00
**Total direct effect**	0.525	0.267	0.000	1.050	33.83
**Total indirect effect**	1.026	0.156	0.743	1.348	66.11
**PP→IADL→CF**	0.917	0.152	0.635	1.230	59.09
**PP→RE→CF**	0.076	0.046	0.003	0.180	4.90
**PP→IADL→RE→CF**	0.034	0.018	0.002	0.071	2.19

*Note*: Physical performance: PP; Instrumental activities of daily living: IADL; Regular exercise: RE; Cognitive function: CF.

## 4 Discussion

This study explored the sequential multiple mediation effects of IADL and regular exercise in the relationship between physical performance and cognitive function among OA‐DM&HTN. The findings revealed that both the combined mediation pathway and individual mediation effects of single mediators demonstrated statistically significant associations.

### 4.1 Comparative Analysis of Demographic Variables for Cognitive Function

The present investigation revealed statistically significant variations in cognitive performance across gender, age, educational background, marital status, and income level among OA‐DM&HTN. These outcomes align with earlier research (Zhang et al. 2024; Aonan et al. 2024). First, male participants generally exhibited better cognitive function than female participants, which may be attributed to biological differences related to gender (e.g., hormone levels and cardiovascular health) (Laws et al. 2016). Males may have a lower risk of neurodegenerative diseases compared to females, which could result in better cognitive performance. Second, age is a crucial factor in cognitive decline; as age increases, neuronal degeneration progressively affects memory, attention, and other cognitive functions (Piccinin et al. 2013). Furthermore, individuals with more years of formal education generally demonstrate enhanced cognitive resilience. This resilience stems from continuous intellectual activity, expertise accumulation, and cognitive challenges, which promote adaptive neuroplasticity (e.g., increased gray matter) and compensatory neural mechanisms. The positive link between higher education and superior cognitive function probably stems from education's role in building cognitive reserve. During education, intellectual activities promote neural plasticity. This helps the brain better withstand age‐related changes, thus boosting brain health and slowing cognitive decline (Piccinin et al. 2013). Furthermore, married patients generally have better social support systems compared to unmarried individuals, which can reduce psychological stress, promote mental and physical health, and positively influence cognitive function (Xiaoshuai et al. 2024; Aonan et al. 2024). Lastly, higher household income typically correlates with a better quality of life and greater access to health resources (e.g., nutrition, healthcare), which can effectively mitigate the negative impact of diabetes and hypertension on cognitive function.

### 4.2 Descriptive Analysis of the Participants’ Physical Function, Instrumental Activities of Daily Living, and Regular Exercise Patterns

The relatively low physical performance scores (mean 1.54 ± 0.78) observed in our sample are consistent with previous research demonstrating that older adults with comorbid diabetes and hypertension experience significant functional decline (Kuo et al. 2005). This finding underscores the vulnerability of this population to physical deterioration and highlights the clinical importance of early intervention strategies. The moderate IADL scores (mean 17.37 ± 6.19) observed in our sample reflect functional challenges in older adults with comorbid diabetes and hypertension. This finding aligns with evidence showing that the coexistence of diabetes and hypertension significantly impacts functional capacity (Ribeiro‐Lucas et al. 2025). Notably, the exercise pattern distribution revealed a concerning trend, with nearly one‐third of participants (31.25%) engaging in no regular exercise, which is particularly problematic given the well‐established benefits of physical activity for managing diabetes, hypertension, and cognitive function. The median exercise frequency of 2 days per week falls below the WHO recommendations (Bull et al. 2020), indicating suboptimal exercise engagement in this high‐risk population. These descriptive findings collectively demonstrate the interconnected nature of physical performance decline, IADL limitations, and suboptimal exercise patterns among OA‐DM&HTN, providing essential context for interpreting the mediation pathways observed in this study.

### 4.3 Association Between Physical Performance and Cognitive Function

One important finding of this study is the confirmation of the hypothesis that physical performance in OA‐DM&HTN can predict cognitive function, with a direct effect of 0.525, explaining 33.83% of the total effect. A previous study (Bortone et al. 2021) has demonstrated that various gait parameters serve as predictors of negative health outcomes across clinical domains, cognitive domains, and physical domains. Moreover, gait characteristics have been established as reliable indicators of overall health status and functional capacity in frail elderly populations. Another study also revealed that reduced gait velocity proved to be the most robust independent predictor of cognitive decline, while additional physical performance metrics also demonstrated significant associations with cognitive impairment (Veronese et al. 2016). In alignment with these previous findings, the current study provides further empirical evidence linking physical function to cognitive status among OA‐DM&HTN.

The observed association may be attributed to several underlying mechanisms. First, good physical performance reflects better cardiovascular health and vascular function, which ensures adequate blood flow and oxygen supply to the brain, thereby supporting normal cognitive function. Second, better physical performance is typically associated with improved metabolic health, including insulin sensitivity and low levels of chronic inflammation—factors that help alleviate the negative impact of diabetes and hypertension on the brain. Furthermore, neurodegenerative processes may lead to declines in both physical and cognitive abilities. Cognitive decline may progress at a slower rate compared to physical deterioration, particularly among individuals with higher educational attainment. Consequently, impaired physical performance represents a risk factor that can potentially be modified for cognitive impairment in OA‐DM&HTN.

### 4.4 The Chain Mediating Effects of Instrumental Activities of Daily Living and Regular Exercise

The findings of this study demonstrate that both IADL and regular exercise function as independent and sequential mediators between physical performance and cognitive function among OA‐DM&HTN. The total indirect effect through these mediators was 1.026, representing 66.11% of the total effect.

Specifically, physical performance was observed to exert an indirect influence on cognitive function through IADL, with an effect value of 0.917. Consistent with recommendations from the WHO, adults are encouraged to engage in at least 150 min per week of MVPA, preferably spread throughout the week, to support sustained exercise behavior and optimize health outcomes. This pathway explains 59.09% of the total mediation effect. OA‐DM&HTN with better physical performance typically exhibits improved gait, balance, and muscle strength, enabling them to engage in more daily activities, participate in social interactions, reduce sedentary behavior, and minimize social isolation—key factors for maintaining cognitive function. A study involving 7,299 older adults, demonstrated that incident, transient, and persistent social isolation raise the risk of cognitive impairment, while persistent loneliness also increases this risk (Huang et al. 2024). Furthermore, good daily activity performance helps reduce emotional issues, such as anxiety and depression, which are often exacerbated by declining physical function and can accelerate cognitive deterioration. Wu et al. demonstrated that older adults with compromised physical health exhibited deteriorated nutritional status, elevated depressive symptomatology, and diminished cognitive function (Wu et al. 2021). Consequently, physical performance may indirectly attenuate the deleterious effects of chronic diseases on neural function and enhance cognitive health through the improvement of IADL.

Furthermore, physical performance can influence cognitive function through regular physical exercise, with an effect size of 0.076, explaining 4.9% of the total effect among OA‐DM&HTN. However, it is important to note that while this mediating effect reached statistical significance, the magnitude is relatively modest and should be interpreted with caution regarding its clinical significance. The underlying mechanisms are likely based on multiple physiological, metabolic, and neurological interactions. Diabetic patients may experience neurotoxicity and microvascular damage due to prolonged hyperglycemia (Liu et al. 2024), while the decreased cerebrovascular elasticity in hypertensive individuals exacerbates cerebral hypoperfusion (Hunt and Cipolla 2024). This dual pathological context causes structural and functional damage to key brain regions, significantly increasing the risk of cognitive impairment. Regular exercise improves cardiovascular health, enhances cerebral blood flow, and increases oxygen supply, thereby promoting neuronal metabolism and functional recovery (Biswas and Prince 2024). Furthermore, physical exercise promotes the release of brain‐derived neurotrophic factors, thereby facilitating neuroplasticity and supporting the functionality of cerebral regions associated with learning and memory processes (Villamil‐Parra and Moscoso‐Loaiza 2024). Simultaneously, regular exercise reduces chronic inflammation and oxidative stress, helping to alleviate neuroinflammation caused by diabetes and hypertension, thus protecting brain function from metabolic and immune perspectives. Despite the small effect size, the clinical importance of regular exercise may be better understood when considering its cumulative benefits and role within the broader mediation framework involving multiple pathways.

This study also found that IADL can influence cognitive function through regular exercise among OA‐DM&HTN. Regular exercise enhances patients' self‐efficacy and provides more opportunities for cognitive stimulation. Furthermore, the psychological benefits of regular exercise, such as reducing anxiety and depression, can indirectly affect cognitive performance. Therefore, daily living abilities serve both as a target for the effects of regular exercise and as an important mediator in promoting cognitive function. This highlights the critical and necessary role of comprehensive interventions among OA‐DM&HTN.

Finally, the chain mediation pathway from physical performance → IADL → regular exercise → cognitive function represents a particularly important sequential mechanism. This pathway suggests that physical performance initiates a cascading process where improved physical capabilities first enhance instrumental activities of daily living, which subsequently facilitate engagement in regular exercise, ultimately benefiting cognitive function. From a theoretical perspective, this sequential relationship reflects the hierarchical nature of functional capacity among OA‐DM&HTN. Physical performance serves as the foundational capacity that enables more complex daily activities; as individuals maintain better balance, gait, and strength, they become more confident and capable of performing instrumental tasks such as shopping, cooking, and managing medications. This enhanced IADL performance, in turn, increases their self‐efficacy and creates opportunities for more structured physical activities and exercise participation. Clinically, this chain mediation pathway has profound implications for intervention design, suggesting that targeting physical performance improvements may trigger a positive cascade effect that sequentially enhances daily functioning, exercise participation, and ultimately cognitive outcomes (Fujita et al. 2025; Ho et al. 2025). This finding supports the development of comprehensive, multi‐component interventions that address physical performance as a primary target while recognizing that the cognitive benefits may be realized through intermediate improvements in daily functioning and exercise behavior, rather than through direct pathways alone.

### 4.5 Implications for Clinical Practice and Future Research

In clinical practice, a comprehensive assessment should be conducted for OA‐DM&HTN, encompassing physical performance, daily living abilities, and cognitive function. Personalized intervention strategies should then be formulated according to the assessment outcomes. Additionally, the design and implementation of these interventions should involve a multidisciplinary team, including endocrinology, cardiology, rehabilitation, and psychology departments. For example, endocrinologists optimize the metabolic management of diabetes and hypertension, rehabilitation specialists improve physical performance and daily function through exercise prescriptions, and psychologists provide cognitive training and psychological support to ensure comprehensive coverage of the intervention's effects. Furthermore, regular exercise should be considered an important component of health management for OA‐DM&HTN. Patients should be encouraged to participate in moderate aerobic exercise (e.g., walking, swimming), resistance training (to enhance muscle strength), and balance exercises (such as Tai Chi or yoga). These intervention strategies should be tailored to the individual's specific health profile to enhance adherence and promote long‐term sustainability of exercise behavior. Concurrently, health education for both patients and their families should be reinforced, as improving cognitive function is a complicated and demanding endeavor. It is essential to raise awareness of the interrelationship between physical performance, activity of daily living, regular exercise, and cognitive function, and to emphasize the importance of lifestyle management. Additionally, personalized intervention platforms utilizing artificial intelligence and mobile health technologies, including wearable sensors and smartphone‐based health applications, warrant investigation for real‐time monitoring of physical performance metrics and daily activity patterns, thereby enabling adaptive modification of intervention protocols and enhancing patient compliance and therapeutic outcomes.

### 4.6 Strengths and Limitations

To our knowledge, this study represents the first empirical effort to investigate the chain of multiple mediating roles of IADL and regular exercise in the association between physical performance and cognitive function among OA‐DM&HTN. Nevertheless, several limitations warrant acknowledgment. The cross‐sectional study design inherently restricts causal determination capabilities. Subsequent research should employ long‐term research methods to validate the sequential mediation pathways through which physical performance influences cognitive function via IADL and regular exercise, elucidating the temporal dynamics and critical transition points within these relationships while providing empirical evidence for intervention optimization. Moreover, although multiple confounding variables were controlled, potential interactions among these covariates were not examined. Additionally, cognitive function was evaluated exclusively using the MMSE, a tool that offers restricted sensitivity in identifying mild cognitive impairment when contrasted with more extensive neuropsychological tests like the Montreal Cognitive Assessment (MoCA). Subsequent research could be enhanced by integrating more nuanced cognitive batteries to better delineate subtle cognitive alterations.

Moreover, physical activity information was obtained via self‐reported questionnaires, which are susceptible to inaccuracies due to memory recall bias and tendencies toward socially desirable responses. While we provided detailed descriptions of exercise modalities and intensity levels, objective monitoring through wearable devices or other objective measures would enhance measurement accuracy and provide more robust evidence for exercise prescription guidelines. Additionally, while we collected data on cardiovascular and cerebrovascular comorbidities, we were unable to obtain comprehensive information on other potential confounding conditions, such as respiratory diseases, musculoskeletal disorders, and other chronic conditions. Future research should incorporate more comprehensive comorbidity assessments to better control for potential confounding factors. Finally, participants were recruited exclusively from two Chinese provinces, limiting generalizability. Subsequent research should incorporate larger, more diverse samples with longitudinal follow‐up to establish causal relationships and extend these findings to broader populations with comorbid chronic conditions.

## 5 Conclusion

In conclusion, this study has made a significant contribution to the understanding of the intricate relationships among physical performance, IADL, regular exercise, and cognitive function among OA‐DM&HTN. Our findings underscore the critical role of physical performance as a key predictor of cognitive function in this population. More importantly, we have unveiled the independent and chain‐mediating effects of IADL and regular exercise in this relationship. This highlights the multifaceted nature of the factors influencing cognitive health in OA‐DM&HTN and provides a more comprehensive picture of the underlying mechanisms. Therefore, to delay cognitive decline among OA‐DM&HTN, it is essential to provide tailored functional training, encourage improvement in IADL, and promote regular exercise among OA‐DM&HTN.

## Author Contributions

Chao Sun, Huixiu Hu, and Xiaoyan Zhang designed the experiment, performed the data analysis and wrote the main manuscript text. Yajie Zhao cleaned the data. Wei Gu designed the experiment and reviewed the manuscript. All authors reviewed the manuscript.

## Funding

This work was supported by the National Natural Science Foundation of China (72304046，72474032), The Beijing High‐Level Innovation and Entrepreneurship Talent Support Program young top talent projects（G202522083），National High Level Hospital Clinical Research Funding (BJ‐2024‐167, BJ‐2024‐199, BJ‐2023‐244), National Key R&D Program of China (2020YFC2008504).

## Ethics Statement

This study was approved by Ethics Committee (approval number: 2021BJYYEC‐325‐01).

## Data Availability

The data that support the findings of this study are available from the corresponding author upon reasonable request.
